# Rapid screening for resistance to *Sitobion avenae* (F.) and *Rhopalosiphum padi* (L.) in winter wheat seedlings and selection of efficient assessment methods

**DOI:** 10.1002/ps.8485

**Published:** 2024-10-18

**Authors:** Ilma A Qonaah, Amma L Simon, Duncan Warner, Rosanna M Rostron, Toby J A Bruce, Rumiana V Ray

**Affiliations:** ^1^ Division of Plant and Crop Sciences, School of Biosciences University of Nottingham Sutton Bonington UK; ^2^ Syngenta UK Ltd. Cambridge UK; ^3^ School of Life Sciences Keele University Keele UK

**Keywords:** *Sitobion avenae*, *Rhopalosiphum padi*, *Triticum aestivum*, aphid resistance, rapid phenotyping, electrical penetration graph (EPG)

## Abstract

**BACKGROUND:**

*Sitobion avenae* (F.) and *Rhopalosiphum padi* (L.) are harmful pests of wheat [*Triticum aestivum* (L.)]. No genetic resistance against the aphids has been identified in commercial wheat varieties and resistance phenotyping can be time‐consuming and laborious. Here, we tested a high‐throughput phenotyping method to screen 29 commercial winter wheat varieties for alate antixenosis and antibiosis. We validated this method using comprehensive behavioural analyses, including alate attraction to volatile organic compounds (VOCs) and a feeding bioassay using an electrical penetration graph (EPG), subsequently highlighting possible sources of resistance.

**RESULTS:**

We observed differences in alate behaviour upon assessing alate settlement on wheat seedlings and attraction towards VOCs, revealing the importance of visual and early post‐alighting cues for alate host selection. Aphid settlement was four times higher on the most preferred variety than on the least preferred variety. Using an EPG bioassay, we identified phloem feeding and stylet derailment parameters linked to resistance. We found antibiosis assessment on detached leaves to be an inadequate screen because it produced results inconsistent with intact leaves assessment. Alate and nymph mortality were identified as key traits signifying antibiosis, showing significant positive relationships with alate reproduction and nymph mean relative growth rate.

**CONCLUSIONS:**

Overall, antixenosis and antibiosis varietal responses were consistent for both aphid species. Alate settlement on wheat seedlings was a more efficient antixenosis screen than an olfactometer assay using VOCs. In addition to assessing alate and nymph survival for antibiosis, this allows for more rapid phenotyping of large numbers of genotypes to identify novel aphid resistance genes for varietal improvement. © 2024 The Author(s). *Pest Management Science* published by John Wiley & Sons Ltd on behalf of Society of Chemical Industry.

## Graphical abstract

Aphid resistance screening on wheat was carried out at three stages: host selection, population growth and feeding activity, where stylet derailment and phloem feeding were linked to aphid resistance.

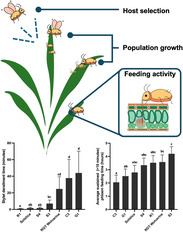

## 1 INTRODUCTION

Aphid pests, along with other arthropods, cause an estimated 18–20% of global crop yield losses.^1^ The English grain aphid [*Sitobion avenae* (F.)] and the bird cherry‐oat aphid [*Rhopalosiphum padi* (L.)] are two of the most economically damaging pests to cereals in Europe.^2^ The aphids remove sap directly from the phloem, transmitting barley yellow dwarf virus (BYDV) and secreting honeydew during feeding, supporting sooty mould growth.^3^ Winter wheat [*Triticum aestivum* (L.)] remains vulnerable to aphid infestations during early tillering, canopy expansion and grain filling, which coincide with increased aphid activity during the flight season.^4^ At present, aphid pests are mainly controlled using insecticides that may have adverse environmental impacts and can result in the evolution of resistance in both *S. avenae* and *R. padi*.^5^, ^6^, ^7^, ^8^ Although integrated pest management (IPM) strategies, such as biological control and intercropping, have been introduced in the past 60 years, they are often suboptimal without insecticide use.^9^ Incorporating aphid resistance into IPM will allow for sustainable pest control with a more stable yield and revenue than insecticide application alone.^10^

Aphid resistance in wheat has been previously identified in ancestral, mutant and landrace lines.^11^, ^12^, ^13^ However, introgressing the desirable resistance genes from these sources by conventional breeding into common wheat can be challenging because of linkage drag of commercially undesirable traits.^14^ Furthermore, development of transgenic crop plants remains restricted under European Union Genetically Modified Organism regulation.^15^ Discovering aphid resistance traits hidden in existing commercial varieties can offer significant advantages for breeders including accelerated varietal improvement without compromising on agronomic traits together with a faster route to market. Rapid screening methods for large plant populations are integral to achieving this purpose.

Host resistance to aphids is differentiated into antixenosis and antibiosis, the former indicated by host avoidance that is commonly phenotyped using whole plant or collected volatile organic compounds (VOCs) choice tests.^16^, ^17^ Antibiosis is assessed by measuring insect mortality and population growth on intact or detached leaves.^16^, ^18^, ^19^ Both phenotyping methods can be time‐consuming and are often unsuitable for rapid, high‐throughput phenotyping of large breeding populations or crosses. Screening in the field using natural aphid infestations is an effective method to evaluate adult plant resistance to *S. avenae*; however, field phenotyping by default is not rapid, with experimentation typically repeated in more than one season, and is subject to interference by predators or environmental factors influencing plant development or aphid incidence. Liu *et al*. screened 94 wheat cultivars for resistance to *S. avenae* using artificial infestation in a greenhouse and natural infection experiments in the field for 2 years and showed that more than 70% of tested wheat cultivars exhibited the same, consistent, resistance responses to *S. avenae* at both seedling and adult plant stages.^20^ These results, in agreement with other published work, suggest that generally seedlings experiments can be used as a proxy identification of resistance against *S. avenae* before further validation in the field using adult plant populations.^21^

Here, our first objective was to develop rapid antixenosis screening for pre‐alighting host traits using whole seedling plants in winged aphid (alate) choice tests. We focused on alate behaviour because these aphids are the first host‐colonisers establishing the aphid population. Specifically, we were focused on leaf resistance as a common trait for both aphid species, *S. avenae* and *R. padi*, because both aphids have been found in the UK and Europe colonising wheat leaves prior to heading (GS51) and these early leaf infestations are particularly important for viral transmission.^22^, ^23^, ^24^, ^25^ During flight, host finding by an alate is driven by chemical and visual cues.^26^ We used olfactometry to measure alate attraction to wheat VOCs, allowing us to separate chemical from visual and post‐alighting cues influencing host selection by *S. avenae* and *R. padi* alates.^17^, ^27^ The host selection phase involves brief probing prior to sustained feeding to determine whether the plant is deemed favourable.^26^ In this work, we used electrical penetration graph (EPG) recordings to characterise aphid probing and feeding behaviour in relation to intrinsic plant characteristics with the aim of narrowing down parameters associated with host acceptance and antibiosis.^28^, ^29^, ^30^ Our last objective was to identify rapidly assessed life‐history traits signifying antibiosis. To achieve this, we assessed and compared a number of aphid life‐history traits in experiments using intact or detached plant leaf material.

## 2 MATERIALS AND METHODS

### 2.1 Plant, aphid resources and growing conditions

A panel of winter wheat [*Triticum aestivum* (L.)] varieties was supplied by Syngenta (Cambridge, UK) and screened for aphid resistance at The University of Nottingham, Sutton Bonington. To protect commercially sensitive information these varieties were designated by a letter and a number as: B1, C1, C2, C3, C4, C5, F1, G1, G2, H1, J1, K1, K2, K3, L1, L2, M1, O1, R1, R2, R3, R5, S1, S3, S4, S5 and T1. RGT Wolverine and Solstice were included as controls because of their wide use and the BYDV resistance in the former variety. Wheat plants were sown in 8 × 12 seed trays (module size: length, 3.5 cm; width, 3.5 cm; depth, 8 cm) using Levington Advance Seed & Modular F2S compost. Following vernalisation for 6 weeks at 4 °C, plants were grown for 2 weeks in a glasshouse maintained at 20 ± 2 °C until they reached the three‐ to five‐leaf stage (GS15). Plants were either used at GS15 for settlement and antibiosis assays or were potted individually in 1‐L pots with John Innes No. 2 compost and grown to mid‐flowering stage (GS65) for volatile collection.

Non‐viruliferous *Sitobion avenae* (F.) and *Rhopalosiphum padi* (L.) obtained from Rothamsted Research (Harpenden, UK) were reared on oats (cv. Gerald) and barley (cv. Valerie) plants in a BugDorm‐4 insect rearing cage (length, 47.5 cm; width, 47.5 cm; height, 47.5 cm) purchased from NHBS Ltd (Devon, UK). The aphids were incubated in a growth room with a 20 °C day temperature, 18 °C night temperature and 16:8 h light/dark photoperiod.

### 2.2 Seedling attraction and settlement assay

All 29 varieties of wheat plants were used at GS15 in these experiments. Two replicates of each variety were randomly arranged in BugDorm‐4D insect handling cages (length, 93 cm; width, 47.5 cm; height, 47.5 cm) purchased from NHBS Ltd. Each plant was potted in one module of a 3 × 4 seed tray (module size: length, 5 cm; width, 5 cm; depth, 6 cm), with a total of five trays per cage. Following 1 h of starvation, 100 alates were released into a cage, and the number of alates that settled on each plant was recorded at 4 h post release. Two experiments were conducted using *R. padi* alates in a growth chamber with a 20 °C day temperature, 18 °C night temperature and 16:8 h light/dark photoperiod. Two experiments using *S. avenae* alate were also carried out. One experiment was performed in a growth chamber with a 20 °C day temperature, 18 °C night temperature and 16:8 h light/dark photoperiod, and the other was conducted in a glasshouse with a temperature of 20 ± 2 °C. Each experiment included at least four cages.

### 2.3 Volatile collection

VOCs were collected from the wheat varieties at mid‐flowering stage (GS65) using a headspace sampling method as described previously.^31^ Before collection, entrainment bags were treated with distilled water and heated at 100 °C for at least 3 h to remove any volatile contaminants. Porapak Q adsorbent filters were used to sample plant volatiles and were conditioned by running 4 mL of dichloromethane through them, followed by heating under the same conditions as the entrainment bags. Wheat heads were covered with an entrainment bag with the inlet airflow line connected through the bottom. A zip tie and polytetrafluoroethylene (PTFE) tape were used to seal the bag and inlet line around wheat stem. The inlet port was set at 500 mL per minute for a constant flow of charcoal‐filtered air. A Porapak Q filter, connected to the outlet line, was inserted into the opening at the top of the entrainment bag and sealed shut with PTFE tape. The outlet port was set at 400 mL per minute and the collection process ran for 24 h. The Porapak Q filters were eluted with 750 μL of dichloromethane and stored at −20 °C in labelled 2‐mL vials. Three replicates were collected from different plants for each variety.

### 2.4 Olfactometer assay

Based on results from the settlement assays on seedlings, six wheat varieties contrasting in their alighting behaviour were selected for further pre‐alighting assessments of *R. padi* alates towards collected wheat VOCs. S3, C3, G1 and RGT Wolverine were chosen for the low alate settlement, S4 for the moderate alate settlement and R1 as a positive control for the high alate settlement observed in the seedling assay. Assessments using *S. avenae* alates were completed beforehand using VOCs of 28 varieties, with R2 excluded because of low germination during vernalisation, and R1 as the control. The choice assay was carried out using a four‐arm olfactometer.^32^, ^33^ Headspace samples of three randomly selected varieties were positioned on three of the olfactometer arms, with R1 as a control on the fourth arm. For each variety, equal parts of three headspace sample replicates were used. Each experiment was repeated ten times with different aphids and olfactometer. To position the headspace sample odour source, a 10‐μL aliquot was deposited on strips of Whatman No. 1 filter paper placed in 6 mL syringe barrels that were connected to the olfactometer as inlet arms. Whatman No. 1 filter paper with a diameter of 110 mm was used to line the olfactometer and air was drawn through the chamber at 200 mL per minute. The number of entries and time the alate spent in each zone were recorded for 16 min using an olfactometer package on RStudio 4.1.2 (RStudio, Boston, MA, USA), with 45° rotation every 2 min to avoid lighting bias in a room with a standardised temperature of 25 °C.^34^, ^35^

### 2.5 EPG feeding bioassay

EPG Giga‐8dd (EPG System, Wageningen, The Netherlands) was used to evaluate aphid feeding behaviour and post‐alighting cue perception following settlement.^30^ This experiment was carried out with C3, G1, S3, S4, R1, RGT Wolverine and Solstice included as an additional control. Apterous *S. avenae* and *R. padi* (5–7 days old) were attached to gold thread (18 μm in diameter) using silver glue, connected to copper wire and soldered onto a brass pin. The main leaf of a plant at GS15 was attached to a glass slide using masking tape to flatten the leaf surface. Aphids were immediately lowered onto the leaf surface after the EPG reading was started. The experiment was set up inside a Faraday cage in a room with a standardised temperature of 25 °C. Aphid feeding was recorded using EPG Stylet+d software for 8 h and annotated using EPG Stylet+a software (EPG System). Previously published EPG experiments have used as low as 8 and more than 12 replicates per treatment to remove variation due to aphid and plant individuals in studies.^36^, ^37^, ^38^ In this work, each variety had ten replicates for each aphid species. Replicates without feeding activity within the first hour, or for three consecutive hours at any point during the reading were discarded.

### 2.6 Antibiosis assay on intact leaves

Two experiments with the 29 varieties, each with four replicates, were performed in a growth chamber, hosting *S. avenae* and *R. padi*, and set up at 20 °C day temperature, 18 °C night temperature and a 16:8 h light/dark photoperiod. Two acrylic tubes of 2.5 cm diameter, 2 cm length and 0.3 cm thickness were used as clip cages to confine aphids on the host. The tubes were glued with 0.5 mm mesh on one end and foam on the other end to minimise leaf injury. One alate aphid was placed inside the cage encompassing the first leaf of GS15 wheat. Afterwards, the following aphid fitness traits were assessed: alate mortality, number and weight of the nymphs produced. One nymph was placed back in the cage and its final weight was recorded 7 days later to determine mean relative growth rate (mRGR), which was calculated as natural log difference in aphid weight over time.^39^

### 2.7 Antibiosis assay on detached leaves

Two experiments using the same varieties characterised in the EPG feeding bioassay were performed with four replicates using just *S. avenae*. These experiments were carried out in 48 mm Petri dishes with 0.5% agar and 10 mg L^−1^ kinetin.^40^ The midsection of the first and main leaves of GS15 plants were cut into 3 cm length and the two leaf sections were placed on the agar. One alate was placed on each leaf sample. Modified plate covers with 0.5 mm mesh glued onto the 1 cm^2^ opening were used to provide airflow. The samples were incubated in a growth room with 21 °C light temperature, 15 °C dark temperature and a 16:8 h light/dark photoperiod for 7 days, after which alate mortality, number and weight of the nymphs produced were recorded. One nymph was placed back on a fresh leaf segment on fresh agar plates of the same wheat variety and its weight recorded after 7 days for mRGR assessment.

### 2.8 Statistical analysis

Statistical analysis was carried out using GenStat 23rd Edition (VSN International, Hemel Hempstead, UK) for all experiments except the EPG bioassay, which was analysed using RStudio 4.2.3 (RStudio). The data from the settlement assay, number of entries to olfactometer zones, alate survival and nymph survival were analysed using a generalised linear model (GLM) with binomial distribution and a logit link function. GLM with Poisson distribution was carried out for count data, such as the number of nymphs. GLM with normal distribution was carried out for continuous data including average nymph weight and mRGR with link function of log or square root. In the GLM, all factors including experimental repeat, day and replicate were included, if significant, in the model. Fisher's least significant difference test at a 0.05 significance level was executed for multiple comparisons.

To analyse the time alates spent in olfactometer zones, log or square‐root transformations were carried out and the normally distributed data were analysed using a two‐sided *t*‐test. Data that did not follow normal distribution despite transformation was analysed using the Mann–Whitney *U*‐test. Linear regression analysis was carried out to investigate the relationship between *S. avenae* alate settlement during the seedling assay and behaviour, namely number of entries during the olfactometer assay, and between parameters observed during antibiosis phenotyping.

Parameters associated with probing (Pr), non‐probing (Np), pathway (C), stylet derailment (F), salivation (E1) and feeding (E2) were obtained from the EPG bioassay. Quasi‐Poisson and gaussian family were applied to GLM analysis of count and continuous data, respectively, with link function log or square root, depending on its residual distribution. Normally distributed data were analysed by analysis of variance. Interactions between wheat varieties, aphid species and time were assessed. Sums of C and E1 parameters were analysed using asymptotic nonlinear regression from the aomisc package (https://rdrr.io/github/OnofriAndreaPG/aomisc/).^41^ Probing activity of *S. avenae* and *R. padi* was analysed using Mann–Whitney *U*‐test.

## 3 RESULTS

### 3.1 Settlement and olfactometer assay

**Figure 1:**
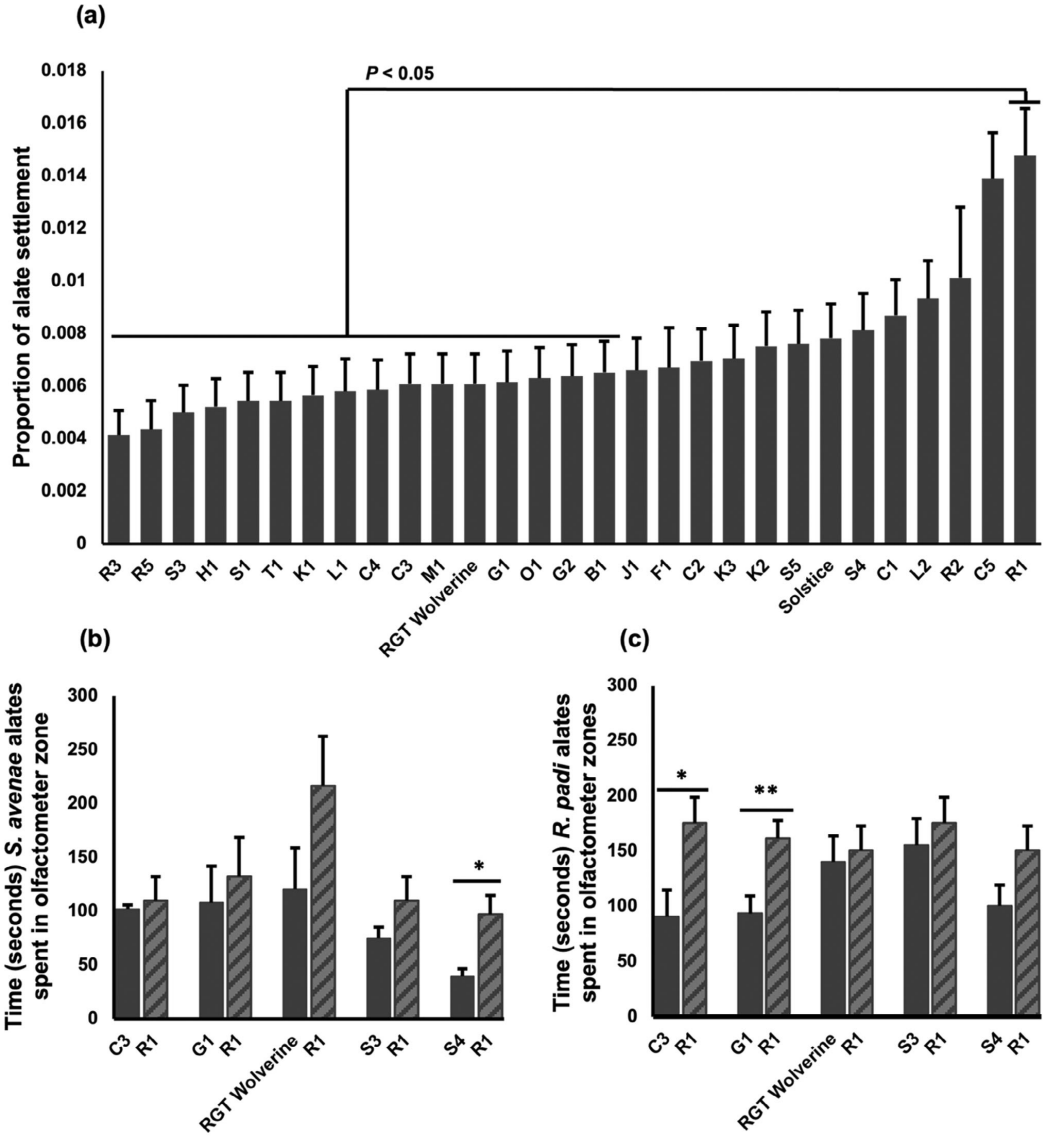
*Sitobion avenae* and *Rhopalosiphum padi* behavioural response to wheat genotypes. (a) Proportion of settled alates analysed using a generalised linear model (GLM) for logistic regression. Data are displayed as back‐transformed means (four experiments). (b) Time *S. avenae* alate spent in olfactometer zones containing the wheat headspace sample, analysed using a *t*‐test (*n* = 10). (c) Time *R. padi* alate spent in olfactometer zones containing the wheat headspace sample, analysed using a *t*‐test and Mann–Whitney *U*‐test (*n* = 10). Pairwise comparisons obtained using Fisher's least significant difference at 0.05 significance. *P ≤ 0.05; **P ≤ 0.01; ***P ≤ 0.001.

The settlement assay on wheat seedlings was designed to provide information on pre‐ and early post‐alighting behaviour of *S. avenae* and *R. padi* alates driving alate settlement on the host. There were no significant differences between aphid species (*P* = 0.181) or interactions among wheat variety, aphid species and experimental repeats (*P* = 0.161), indicating that varietal effects on alate settlement were consistent across experiments and both aphid species. A significantly greater number of alates of *S. avenae* and *R. padi* settled on R1, identified as the most attractive variety, than on B1, G2, O1, G1, RGT Wolverine, M1, C3, C4, L1, K1, T1, S1, H1, S3, R5 and R3 (*P* < 0.001; Fig. 1(a)).

To separate the contribution of chemical cues from that of visual cues on alate settlement behaviour, we carried out olfactometer assays using collected wheat volatiles. We focused on alate attraction responses, indicated by the time spent in olfactometer zones with VOCs of the most attractive variety, R1, identified in the seedling assay, as a positive control for the assessment of G1, S3, C3 and RGT Wolverine that were found significantly less attractive by alates, in addition to Solstice and S4. Alates of *S. avenae* spent significantly less time on S4 than on R1 (*P* = 0.043; Fig. 1(b)), but not on other varieties, whereas *R. padi* alates spent significantly less time on G1 (*P* = 0.003; Fig. 1(c)) and C3 than on R1 (*P* = 0.015; Fig. 1(c)). There were no differences in alate attraction between R1 and S3 or RGT Wolverine, despite low alate settlement in the seedling assay on the latter two varieties, suggesting that chemical cues alone were not driving settlement on these genotypes.

**Figure 2:**
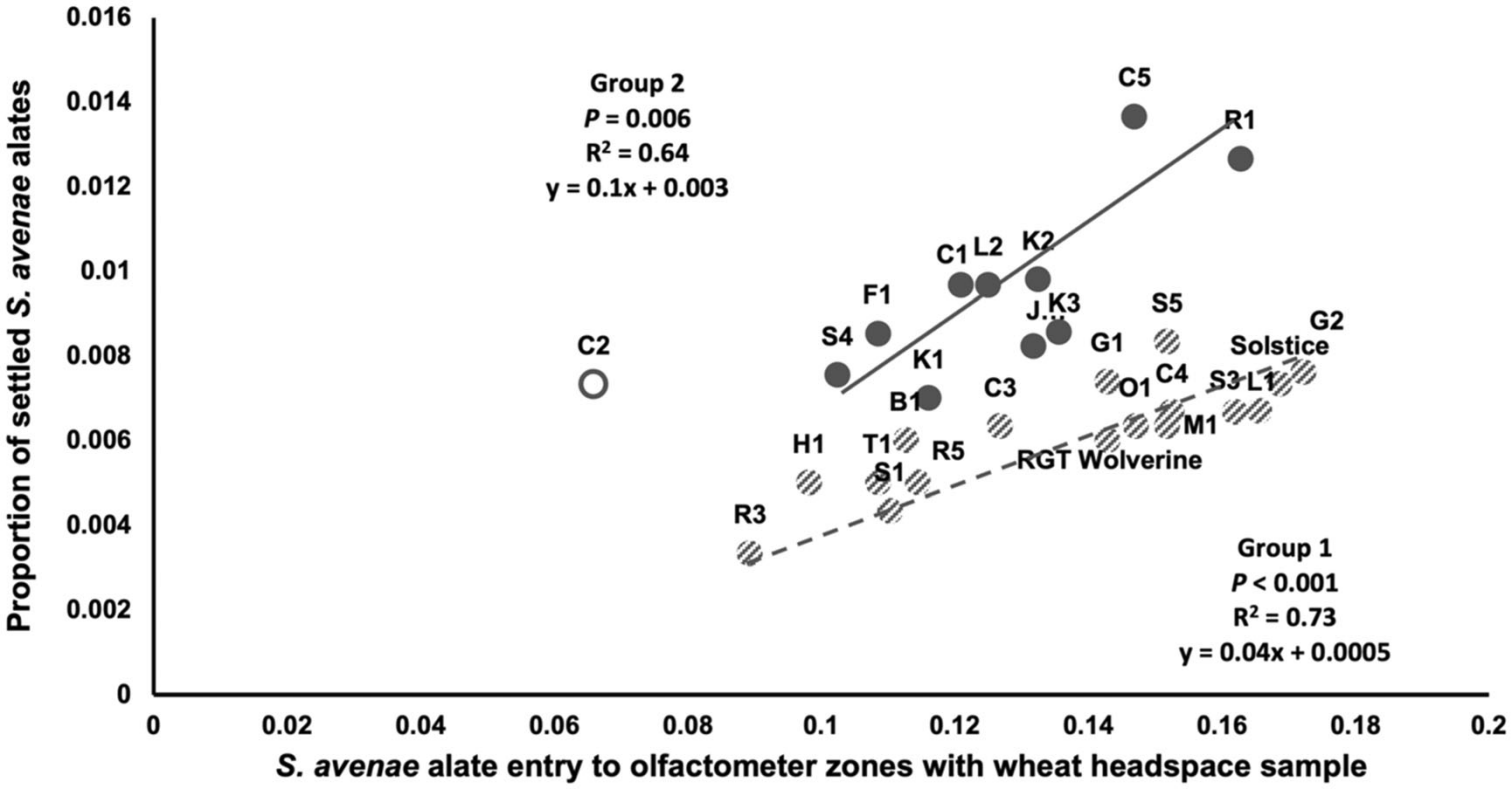
Relationships between alate settlement in the seedling assay and number of entries of alate of *Sitobion avenae* into olfactometer zones with wheat headspace samples determined by linear regression analysis. Data fitted two separate lines identifying group 1 (*R* ^2^ = 0.73, *y* = 0.04*x* + 0.0005, *P* < 0.001) and group 2 (*R* ^2^ = 0.64, *y* = 0.1*x* + 0.003, *P* = 0.006) of varieties. C2 was excluded from analysis as an outlier.

Linear regression of *S. avenae* alate settlement on the number of entries in the olfactometer assay showed significant positive relationships between the two variables with data for all varieties fitting two separate lines identifying two varietal groups: group 1 (*R* ^2^ = 0.73, *P* < 0.001; Fig. 2) and group 2 (*R* ^2^ = 0.64, *P* = 0.006; Fig. 2). Although varieties within both groups generally elicited similar attraction behaviour based on VOCs cues alone, varieties from group 1, inclusive of G1, S3, C3, RGT Wolverine and Solstice, appeared to be less visually attractive to alates of both aphids in the seedling assay than varieties of group 2, inclusive of R1 and S4. C2 was identified as an outlier because of unattractive volatiles but moderate alate settlement. These results suggested that the seedling assay was a comprehensive method for rapidly assessing antixenosis because alate settlement of both aphid species appeared driven by combined visual and chemical cues.

### 3.2 Aphid feeding activity

To characterise aphid feeding behaviour following settlement, we carried out EPG assays with the same varieties used for olfactometry studies and Solstice. A summary of significant parameters, main effects and interactions is shown in Supporting Information, Table S1. Probing activity of *S. avenae* and *R. padi* was consistent on the assessed varieties, showing no significant interactions between aphid species and wheat varieties for number of parameters, including time of probing, pathway, stylet derailment and sustained feeding phases (Supporting Information, Table S1).

**Figure 3:**
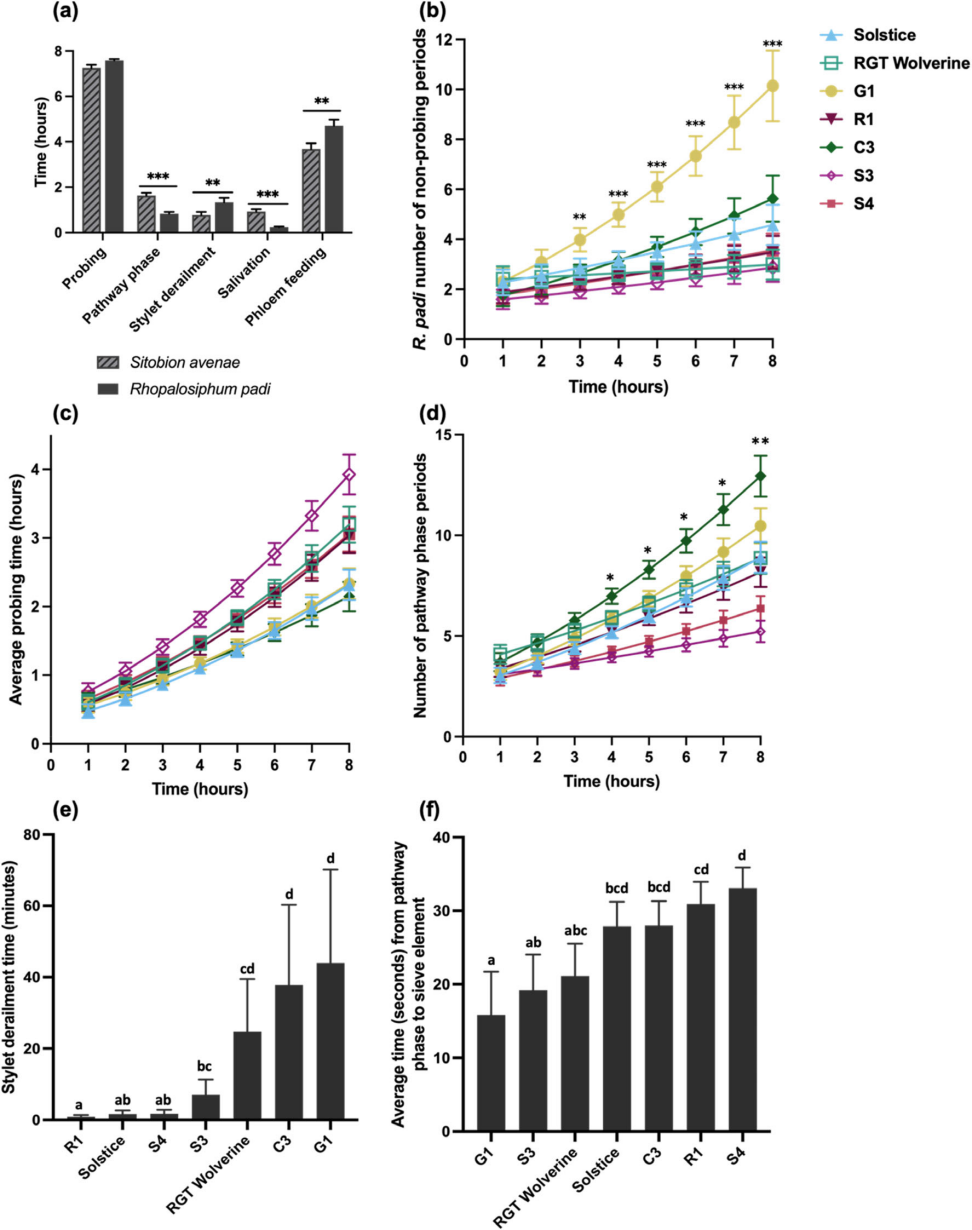
Electrical penetration graph readings of *Sitobion avenae* and *Rhopalosiphum padi* on Solstice, G1, R1, RGT Wolverine, C3, S3 and S4 with data displayed as back‐transformed means. (a) Comparison of *R. padi* and *S. avenae* probing activity, including pathway phase (*P* < 0.001), stylet derailment (*P* = 0.008), salivation (*P* < 0.001) and phloem feeding (*P* = 0.003), analysed using Mann–Whitney *U*‐test (*n* = 20). (b) Number of non‐probing phases for *R. padi* on wheat varieties over 8 h (*P* = 0.022), analysed using a generalised linear model (GLM) with Poisson distribution and link function of square root (*n* = 10). (c) *R. padi* and *S. avenae* average probing time on wheat varieties over 8 h (*P* = 0.008), analysed using gaussian distribution with link function of square root (*n* = 20). (d) *R. padi* and *S. avenae* number of pathway phases on wheat varieties over 8 h (*P* < 0.001), analysed using Poisson distribution with link function log (*n* = 20). (e) *R. padi* and *S. avenae* stylet derailment times on wheat varieties (*P* = 0.027) analysed using GLM normal distribution with link function log (*n* = 20). (f) *R. padi* and *S. avenae* average time from mesophyll (pathway phase) to sieve element on wheat varieties (*P* < 0.001) analysed using GLM with normal distribution and square root link function (*n* = 20). *P ≤ 0.05; **P ≤ 0.01; ***P ≤ 0.001.

We identified differences between aphid species for feeding behavioural strategies on leaves of wheat varieties (Fig. 3(a)). Thus, *S. avenae* spent longer in the pathway phase (*P* < 0.001; Fig. 3(a)) to reach sieve elements and to salivate compared with *R. padi* (*P* < 0.001; Fig. 3(a)). By contrast, *R. padi* exhibited longer periods of stylet derailment than *S. avenae* (*P* = 0.008; Fig. 3(a)). However *R. padi* also sustained a longer period of feeding from the phloem than *S. avenae* (*P* = 0.003, Fig. 3(a)).

**Figure 4:**
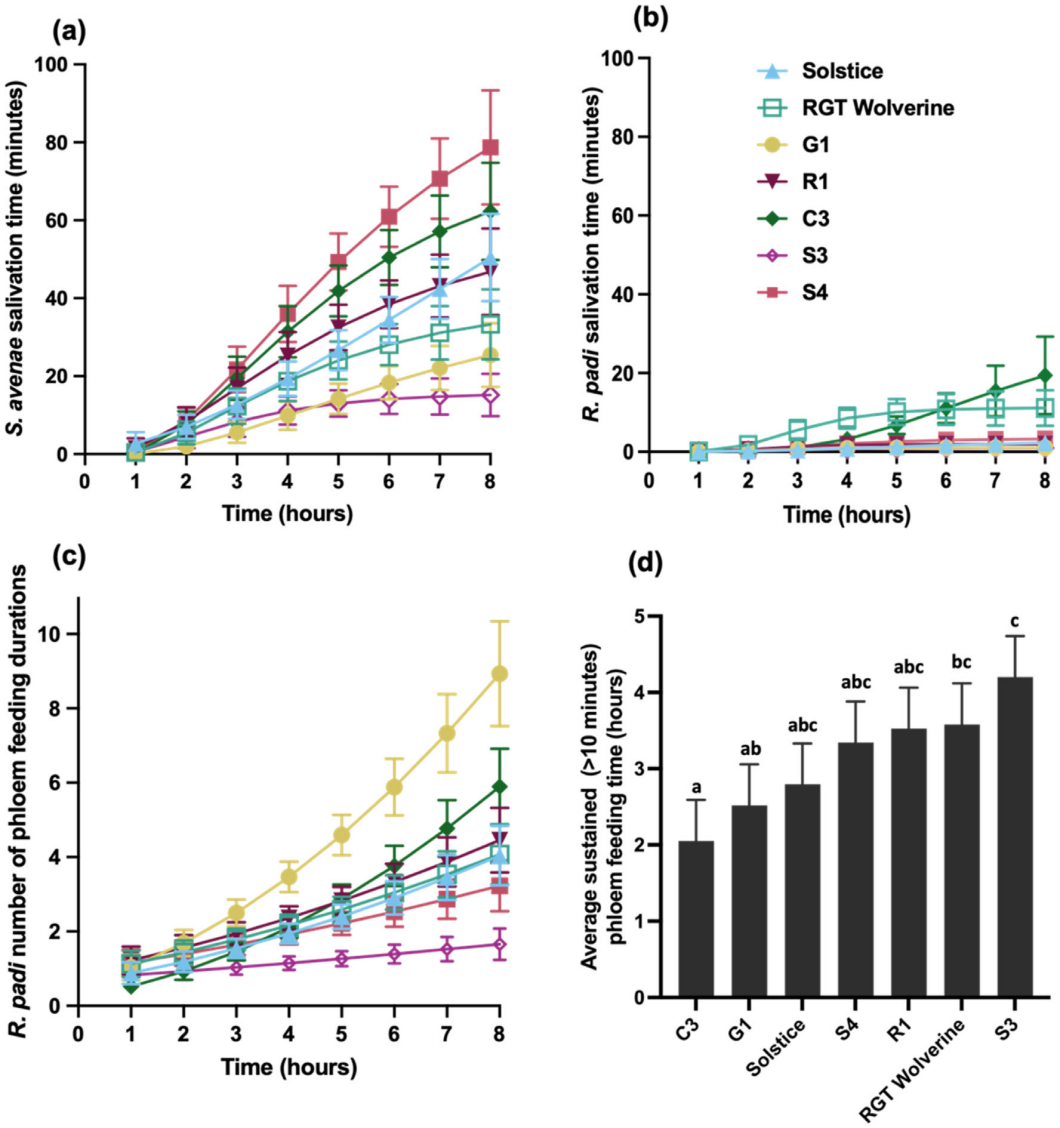
Electrical penetration graph readings of *Sitobion avenae* and *Rhopalosiphum padi* on Solstice, G1, R1, RGT Wolverine, C3, S3 and S4 with data displayed as back‐transformed means. (a) *S. avenae* salivation time on wheat varieties over 8 h (*P* < 0.001), analysed using asymptotic nonlinear regression (*n* = 10). (b) *R. padi* salivation time on wheat varieties over 8 h (*P* < 0.001), analysed using asymptotic nonlinear regression (*n* = 10). (c) *R. padi* number of phloem feeding phases on wheat varieties over 8 h (*P* = 0.003), analysed using a generalised linear model (GLM) with Poisson distribution with link function square root (*n* = 10). (d) *R. padi* and *S. avenae* average, sustained (>10 min), feeding time on wheat varieties (*P* = 0.024) analysed using GLM with normal distribution with link function log (*n* = 20). *P ≤ 0.05; **P ≤ 0.01; ***P ≤ 0.001.

Individual aphid–varietal interactions were revealed from 3 h following aphid introduction onto the host, with *R. padi* spending increasingly longer non‐probing periods on G1 than on any other variety (*P* = 0.022; Fig. 3(b)). Differences in aphid probing and pathway periods were detected between varieties at 4 h following aphid settlement showing that both *S. avenae* and *R. padi* spent less time probing (*P* = 0.008; Fig. 3(c)) with an increased number of pathway phase periods on G1 and C3 than on S3 (*P* < 0.001; Fig. 3(d)). These two observations for the aphids were consistent with the significantly increased stylet derailment measured on C3 and G1 (*P* = 0.027; Fig. 3(e)) suggesting increased obstructions for aphids going through the mesophyll layer of these varieties. Both aphids spent least time on G1 and S3 to reach the sieve elements from the pathway phase compared with C3, R1 and S4 (*P* < 0.001; Fig. 3(f)). We observed significant differences between aphid species and variety for salivation time (Fig. 4(a,b)). Increased salivation from 5 h of the EPG recording was observed for *S. avenae* on S4 and C3 compared with G1 and S3 (*P* < 0.001; Fig. 4(a)). *R. padi* salivated first and most on RGT Wolverine and C3 and least on G1 (*P* < 0.001; Fig. 4(b)). Differences in the number of phloem feeding events were observed only for *R. padi* showing the highest number phloem feeding periods on G1 over time compared with S3 (*P* = 0.003; Fig. 4(c)). Ultimately, aphids experienced most difficulties sustaining phloem feeding of more than 10 min on C3 and G1, in contrast to S3 or RGT Wolverine where both aphids spent, on average, 3.5–4 h in sustained feeding (*P* = 0.024; Fig. 4(d)).

### 3.3 Antibiosis screening

We first carried out *S. avenae* antibiosis traits screening on detached or intact leaves to investigate the consistency of varietal evaluation when using detached plant material or material with an intact defence response, and to identify key traits that can be assessed more rapidly. Overall, the assessment of *S. avenae* life‐history traits on detached leaves failed to correlate with results from the intact leaves assessment (Supporting Information, Fig. S1). We observed antibiosis in the form of low *S. avenae* alate survival (*P* = 0.021; Supporting Information, Fig. S1(a)) and fecundity (*P* < 0.001; Supporting Information, Fig. S1(b)) on C3 and G1, but only on intact leaves. Furthermore, we found significant interactions between experimental repeats and varietal responses for fecundity assessments on detached leaves (*P* < 0.001; Supporting Information, Fig. S1(b)) with no significant differences between varieties (*P* = 0.38; Supporting Information, Fig. S1(b)). By contrast, *S. avenae* mRGR was significantly different only on detached wheat leaves (*P* = 0.021; Supporting Information, Fig. S1(c)) and not on intact leaves (*P* = 0.37; Supporting Information, Fig. S1(c)), with opposing trends determined by the two methods.

**Figure 5:**
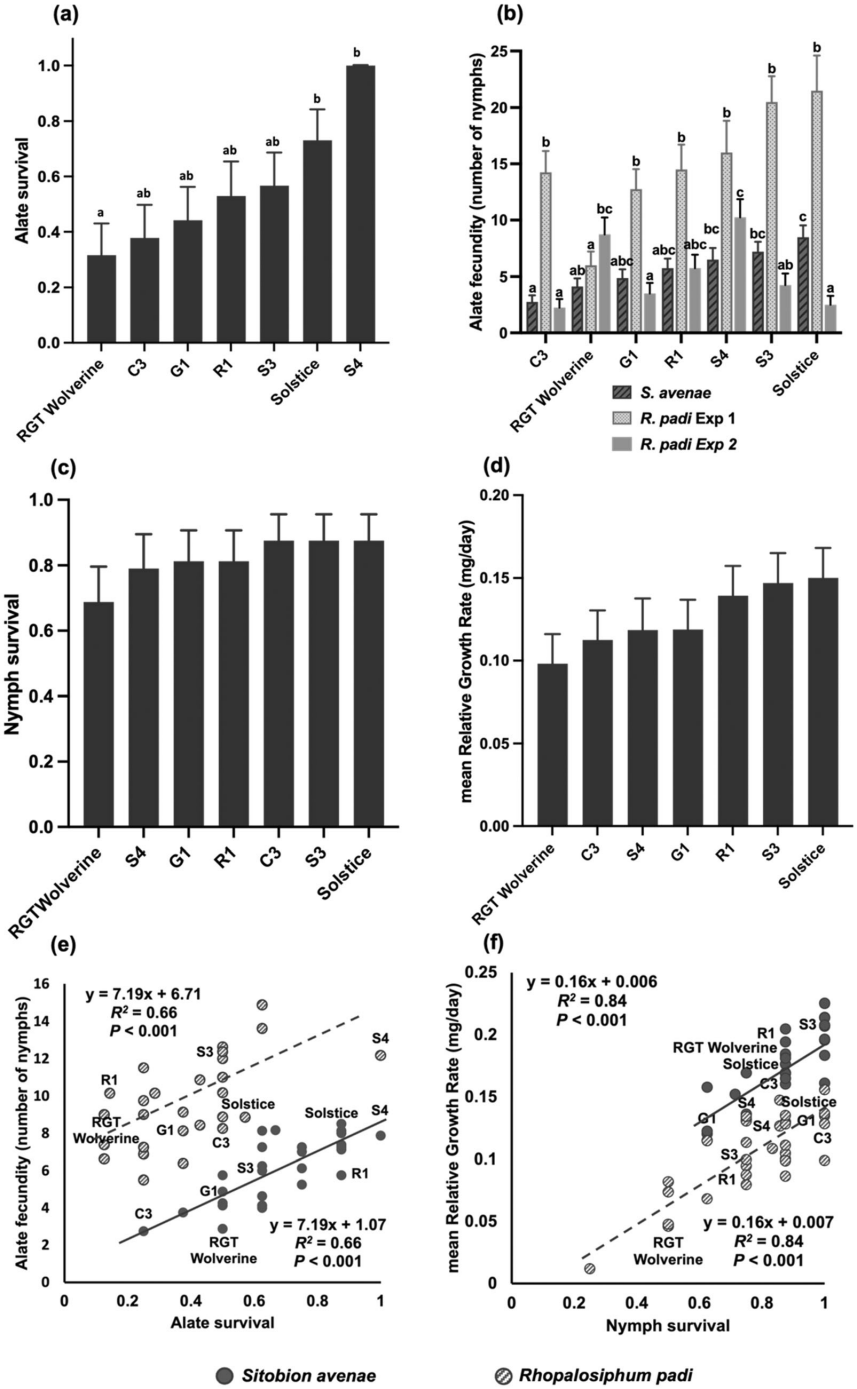
*Sitobion avenae* and *Rhopalosiphum padi* antibiosis assessment on intact leaves analysed using a generalised linear model (GLM) with data displayed as back‐transformed means (*n* = 16). (a) *R. padi* and *S. avenae* alate survival on wheat varieties (*P* < 0.001), analysed with binomial distribution and logit link. (b) *R. padi* alate fecundity produced a significant interaction between experimental repeat and wheat varieties (*P* = 0.006). Data analysed using Poisson distribution with square root link. (c) *S. avenae* and *R. padi* nymph survival did not exhibit differences for wheat varieties (*P* = 0.83), analysed using GLM logistic regression with logit link function. (d) *S. avenae* and *R. padi* mRGR did not exhibit a difference for wheat varieties (*P* = 0.84), analysed using GLM normal distribution with link function log. (e) Simple linear regression with groups for *S. avenae* and *R. padi* alate survival and fecundity showing significant positive relationships with data fitting two parallel lines (*P* < 0.001). (f) Simple linear regression with groups for *S. avenae* and *R. padi* nymph survival and apterous mRGR showing significant positive relationships with data fitting parallel lines (*P* < 0.001). Pairwise comparisons obtained using Fisher's least significant difference at 0.05 significance.

Because of the observed inconsistencies in *S. avenae* life‐history traits determined on detached and intact wheat leaves, we omitted assessments on detached leaves using *R. padi*. Using intact leaves experiments, we found alate survival (*P* < 0.001; Fig. 5(a)) to be the most consistent parameter, showing the lowest survival rate on RGT Wolverine (32 ± 11%), followed by C3 and G1, and the highest survival on S4 (100%) and Solstice (73 ± 11%). There was no significant interaction among wheat varieties, aphid species and experimental repeats for alate survival of *R. padi* and *S. avenae* indicating that this trait can be used consistently for antibiosis assessment for both aphid species.

Unlike the intact leaves experiment using *S. avenae*, experiments with *R. padi* were less consistent, showing interaction between varieties and experimental repeats (*P* < 0.001; Fig. 5(b)) for alate fecundity. The easily identified inconsistencies between experimental repeats were associated with performance on RGT Wolverine and Solstice, which contrasted for number of nymphs from alate in each experiment. There were no significant differences between varieties for nymph survival (*P* = 0.83; Fig. 5(c)) or mRGR (*P* = 0.84; Fig. 5(d)) of *S. avenae* or *R. padi*.

To identify significant relationships between antibiosis traits we performed linear regression analysis with groups for each aphid species. There was a significant positive relationship between alate survival and fecundity, accounting for 66% of the variance with the data fitting parallel lines with the same slope but different intercepts for *S. avenae* and *R. padi* (*P* < 0.001; Fig. 5(e)). This showed that intrinsic alate fecundity is higher in *R. padi* than in *S. avenae*. Nymph survival was also positively related to mRGR fitting parallel lines with 84% of variance accounted for in *S. avenae* and *R. padi* (*P* < 0.001; Fig. 5(f)), with *S. avenae* displaying an increased, narrow range of survival on the assessed varieties with higher mRGR of survived nymphs than *R. padi*.

## 4 DISCUSSION

Host colonisation by aphids is mainly achieved by alates because of their ability to fly as far as 100 m, whereas apterous aphids are only able to move over short distances.^42^ However, alates do not possess strong directional control over their flight path, with as few as <1 in 100 *R. padi* alates managing to colonise new hosts in the field.^43^, ^44^ Under field conditions, abiotic factors such as high temperature and wind speed can impede alate flight.^26^ This raises the question of whether pre‐alighting cues have significant impact on alate settlement or whether settlement is purely by chance. This study removed the environmental factors, allowing alates to make host selections under prime conditions. Of the 100 released alates, we observed on average 38 settling after 4 h, with both *R. padi* and *S. avenae* alates demonstrating clear settlement preferences towards R1, confirming a behavioural response towards pre‐ and early post‐alighting cues.

Our observation of alate attraction towards wheat VOCs further affirmed the role of chemical cues in alate host selection, because we observed *S. avenae* and *R. padi* alates attraction to R1 volatiles. Aphids possess olfactory receptors located in their antennae and chemosensory system, although genetic control varies in aphid species depending on their host and life cycle.^45^, ^46^ As a host‐alternating aphid, *R. padi* relies on its olfactory senses to mate and migrate, because its sexual female and male morphs are produced separately on its primary and secondary hosts, respectively.^47^ This necessity is absent in *S. avenae*, a non‐host‐alternating aphid. This perhaps explains the higher sensitivity in the behavioural response of *R. padi* compared with *S. avenae* observed during the olfactometer bioassay, with *R. padi* aversion to C3 and G1, varieties exhibiting low alate settlement, as opposed to *S. avenae* aversion to S4, a variety with moderate alate settlement.

Here we demonstrated the contribution of visual and early post‐alighting cues towards the variation explaining different groupings of the assessed genotypes from the seedling and olfactometer assays. Regression analysis confirmed two distinguishable groups of varieties with group 2 possessing more attractive visual and early post‐alighting cues than group 1. This is exemplified by *S. avenae* low attraction to S4 volatiles from group 2, despite high settlement during the seedling assay. Moreover, RGT Wolverine from group 1 exhibited antixenosis during the settlement assay but demonstrated higher attraction in olfactometer studies. The overall conclusion from these results is that to accurately assess alate antixenosis, the contribution of visual cues should be considered part of the phenotyping screen.

There is a lack of understanding of the way the aphid perceives visual cues, largely due to the ultraviolet (UV) receptor, in addition to blue and green, in its photoreceptor system.^48^ The maximum sensitivity of the aphid photoreceptor is towards the green–yellow colour spectrum to assist the aphid in finding host plants.^48^ Specific wavelength sensitivity varies with aphid species.^26^, ^49^ For instance, Kieckhefer *et al*. demonstrated *R. padi* showed preference for the colour yellow, which has been linked to the attraction of this aphid to BYDV‐infected plants, whereas *S. avenae* displayed more affinity to green.^50^ However, more recent research has not detected any difference in colour preference between aphid species.^51^ Our findings corroborated this, because we did not find a behavioural difference in *S. avenae* and *R. padi* alates during the settlement assay. This may suggest a similarity in the visual preference of the two aphid species or that a post‐alighting cue has bigger role in alate settlement than a visual cue. Furthermore, it would be useful to confirm results using viruliferous aphids because the preference of these aphids may be different under the influence of a virus.

In the field, an alate aphid frequently lands on a plant, samples the plant during a brief probing period, takes flight and repeats this process until a suitable host is found.^42^ The post‐alighting signals alates receive at this stage make the difference between host location and settlement. Here, we highlight the consistency between *S. avenae* and *R. padi* feeding behaviour, with short average probing time, a high number of pathway phases, extensive stylet derailment time and relatively shorter sustained phloem feeding time on C3 and G1 than on other varieties. These traits point to the aphid stylet's difficulty in accessing and staying in the phloem of C3 and G1, and might be the reason behind low alate settlement. This is in line with a previous study by Escudero‐Martinez *et al*. in which a possible resistance factor in the phloem was associated with low settlement.^52^ Phloem sap composition and sieve element occlusion by callose are possible explanations, causing the stylet to retract to the mesophyll, leading to high number of pathway phase events.^53^, ^54^, ^55^ In addition, we found a possible resistance factor in the mesophyll, evidenced by the high stylet derailment period, to also cause low settlement because we observed higher stylet derailment time on S3 and RGT Wolverine, varieties exhibiting lower settlement, than on R1 despite not displaying resistance in the phloem. The mechanisms behind stylet derailment are less known, although plant cell wall composition has been speculated to play a role.^30^, ^56^ Future research should focus on identifying potential resistance factors in the phloem and mesophyll of G1 and C3 using omics approaches. It is possible that digestion inhibitors, changes in nutrient levels or the presence of toxic antibiotic compounds are behind these resistance traits.

There were a few differences between *S. avenae* and *R. padi* probing behaviour, particularly in the number of non‐probing and feeding events where *R. padi* displayed interaction with wheat varieties and time, whereas *S. avenae* did not. Furthermore, we found overall salivation time to be significantly higher for *S. avenae* than for *R. padi*. The aphids secrete watery saliva to suppress sieve element occlusion, with transmission of BYDV also taking place during salivation.^57^, ^58^ Increased salivation time by *S. avenae* may indicate a greater capacity to transmit BYDV into a host compared with *R. padi*. Furthermore, the observed moderate to high salivation time on the BYDV‐resistant RGT Wolverine in relation to other varieties suggested that the resistance is unlikely to operate against viral transmission by the vectors although further investigation using viruliferous aphids is required to test both hypotheses.

Our assessment of *S. avenae* life‐history traits on detached leaves failed to detect resistance in the form of alate survival and fecundity found in intact leaves. This supports previous studies in which the detached leaf method failed to detect resistance to aphids and pollen beetle.^59^, ^60^ The loss of resistance in detached leaves suggested that an inducible defence response is likely required for callose deposition, or constitutive metabolic expression involved in pre‐formed defence that prevents damage on intact leaves.^61^ Moreover, loss of turgidity in detached leaves may also cause its inaptness in phenotyping antibiosis.^62^

In the intact leaves experiments, alate survival and nymph survival were significantly and positively correlated with fecundity and mRGR, respectively. The host colonisation strategy by *R. padi*, in contrast to *S. avenae*, was characterised by high alate fecundity compensating for low alate survival. The high fecundity rate in *R. padi* is likely to have evolved based on its need to migrate to a secondary host, where only small percentage of aphids would survive and successfully colonise the host plant.^44^ The coexistence of morphs makes it difficult to isolate one in an experiment and is possibly the reason behind the discrepancy we saw in *R. padi* alate fecundity between experimental repeats.^63^ In contrast to *R. padi*, host colonisation by *S. avenae* was characterised by higher mRGR of survived nymphs. These results suggest that alate survival and fecundity are important antibiosis parameters for *R. padi*, whereas nymph survival and mRGR of nymphs are important traits for antibiosis for *S. avenae*. Aphid fecundity has been previously linked to phloem feeding disruption.^64^ Our findings validated this, because aphids spent less time feeding on C3 and G1, consistent with the relatively low fecundity and survival on intact leaves of both varieties. However, it is also clear from our results that antibiosis resistance to alates and apterous morphs may differ significantly, and the varieties we have identified as resistant possess antixenosis and antibiosis traits that function against alates because varietal differences in nymph life‐history traits were not significantly different for the assessed genotypes.

In conclusion, we established methods to rapidly screen antixenosis and antibiosis against both aphid species on wheat in seedlings. Alate settlement using the seedling assay allowed for more rapid antixenosis screening incorporating responses to visual and chemical cues while also simulating as close as possible host colonisation under field conditions. For antibiosis, focusing on more rapidly assessed alate and nymph survival traits can be suitable options for high‐throughput screening of large populations under glasshouse conditions, because these traits are representative of alate fecundity and nymph mRGR. We identified potentially resistant varieties C3 and G1 and RGT Wolverine, a BYDV‐resistant variety, to alate antixenosis and antibiosis to both aphid species possessing resistance factors in the phloem and mesophyll. Validation of head antibiosis in the field using adult plant populations is required, specifically with *S. avenae* that is best adapted to feed on wheat heads. Future research includes crossing of the identified varieties with the susceptible variety R1 to identify quantitative trait loci associated with stylet derailment and short phloem feeding. Furthermore, investigating BYDV transmission on these varieties would give us an insight into any BYDV resistance mechanisms and whether these are linked to alate aphid resistance.

## FUNDING INFORMATION

IAQ is a recipient of Biotechnology and Biological Sciences Research Council iCASE studentship program funded by UK Research and Innovation (grant number BB/T008369/1) in collaboration with Syngenta.

## CONFLICT OF INTEREST

The authors declare that they have no conflict of interest.

## AUTHOR CONTRIBUTIONS

IAQ designed and performed the experiments, analysed the data and wrote the manuscript. ALS assisted with experiment design and data analysis for olfactometer assay and EPG feeding assay. RMR performed the detached leaves assays and an experimental repeat of seedling assay. DW and TJAB reviewed and commented on the manuscript. RVR acted as the main advisor for the work and assisted in writing the manuscript. All authors contributed, read and approved the manuscript.

## Supporting information


**Data S1.** Supporting Information.

## Data Availability

The data that support the findings of this study are available from the corresponding author upon reasonable request.
