# Supplementary material for: Rapid screening for resistance to Sitobion avenae (F.) and Rhopalosiphum padi (L.) in winter wheat seedlings and selection of efficient assessment methods
Source: Pest Manag Sci. 2024 Oct 18;81(2):819–30. doi: 10.1002/ps.8485 (PMC11716336; doi:10.1002/ps.8485)
Supplement: Supplementary file 1 — Data S1. Supporting Information. [file PS-81-819-s001.docx]

| **Parameter** |  | **Analysis** | **Variety** | **Aphid** | **Variety*Aphid** | **Variety*Time** | **Definition** |
| --- | --- | --- | --- | --- | --- | --- | --- |
| n_Np | *R. padi* | GLM (sqrt) | - | N/A | N/A | * | Number of non-probing |
| a_Pr |  | GLM (sqrt) | * | - | - | ** | Average probing period |
| n_C |  | GLM (sqrt) | ** | - | - | *** | Number of pathway phase period |
| s_F |  | GLM (log) | * | - | - | - | Sum of stylet derailment time |
| at_C_1E_Pr |  | ANOVA (sqrt) | *** | *** | - | N/A | Average time from pathway to first sieve element |
| s_E1 | *R. padi* | Non-linear regression | *** | N/A | N/A | *** | Sum of salivation time |
|  | *S. avenae* | Non-linear regression | ** | N/A | N/A | *** |  |
| n_qE2 | *R. padi* | GLM (sqrt) | - | N/A | N/A | ** | Number of phloem feeding |
| a_sE2 |  | GLM (log) | * | - | - | - | Average sustained feeding period |

**Table S1**: Electrical Penetration Graph (EPG) bioassay parameters analysis using Generalised Linear Model (GLM), ANOVA, and non-linear regression.


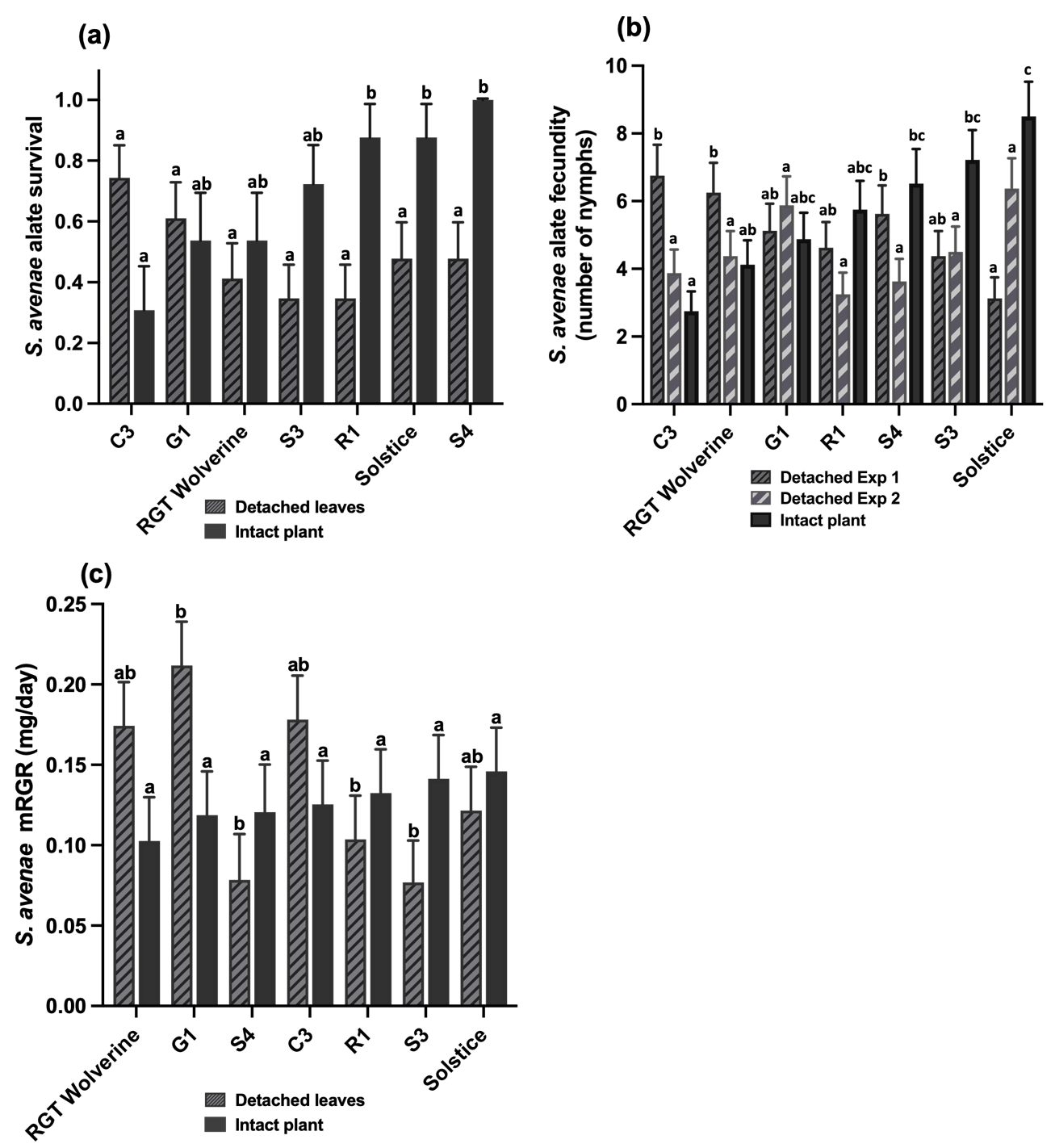


**Figure S1**. *Sitobion avenae* antibiosis assessment on detached and intact leaves assessed using Generalised Linear Model (GLM) with data displayed as back-transformed means (*n* = 16). (a) Alate survival differed significantly (*P* = 0.012) on wheat varieties using intact plant method, but not detached leaves (*P* = 0.28), data analysed using binomial regression with logit link. (b) Alate fecundity differed significantly (*P* < 0.001) on wheat varieties using intact plant method, while detached leaves method produced interaction between experiment (*P* < 0.001) and wheat variety, showing no significant differences for wheat varieties (*P* = 0.38), data analysed using Poisson distribution with square root link. (c) Apterous mRGR differed significantly (*P* = 0.021) on detached leaves but not on intact plants (*P* = 0.37), analysed using GLM with normal distribution and log link. Pairwise comparison obtained using Fisher LSD at 0.05 significance.
